# Pharmacological targeting PIKfyve and tubulin as an effective treatment strategy for double-hit lymphoma

**DOI:** 10.1038/s41420-022-00833-9

**Published:** 2022-01-28

**Authors:** Liying Feng, Kai Chen, Wei Huang, Yuelong Jiang, Xihuan Sun, Yong Zhou, Li Li, Yin Li, Xianming Deng, Bing Xu

**Affiliations:** 1 Department of Hematology, The First Affiliated Hospital of Xiamen University and Institute of Hematology, School of Medicine, Xiamen University, 361003 Xiamen, China; 2 The First People’s Hospital of Foshan (The Affiliated Foshan Hospital of Sun Yat-sen University), 528000 Foshan, Guangdong China; 3 State Key Laboratory of Cellular Stress Biology, Innovation Center for Cell Signaling Network, School of Life Sciences, Xiamen University, 361003 Xiamen, China; 4 Department of Oncology, The First Affiliated Hospital of Jinan University, Jinan University, 510630 Guangzhou, China

**Keywords:** Targeted therapies, Targeted therapies

## Abstract

Double-hit lymphoma is one of the most aggressive and refractory lymphoma subtypes with recurrent genetic abnormalities of MYC and BCL-2 or BCL6 rearrangement, leading to a poor prognosis in the present clinical practice. Therefore, new therapeutic strategies for eliminating double-hit lymphomas are urgently needed. Here, we reported that HZX-02-059, a novel PIKfyve and tubulin dual-target inhibitor, showed a highly cytotoxic activity against double-hit lymphoma cell lines in vitro and in vivo through a noncanonical caspase-independent cell death, methuosis. Mechanistically, the cytotoxicity triggered by HZX-02-059 was contributed to the PIKfyve/TFEB axis-induced cell death of methuosis, as well as the inhibition of tubulin and mTOR/Myc axis-induced cell cycle arrest. In summary, the present findings suggest that HZX-02-059 represents a good starting point for developing targeted therapeutics against double-hit lymphomas.

## Introduction

Double-hit lymphoma (DHL) represents a distinct entity with the acquisition of MYC and BCL-2 or BCL6 translocations, and with a dismal prognosis after standard immunochemotherapy [1]. The dysregulation of these driving oncogenes confers tumor cells uncontrolled proliferation and disordered cell death [2]. Even though the prognosis of the majority of non-Hodgkin lymphomas (NHLs) have significantly improved in the past decades, DHL remains highly resistant to the standard chemoimmunotherapy with an extremely poor clinical outcome of a 30% 5-year overall survival rate [3]. In addition, the present first-line strategy also brings some serious side effects, increasing the treatment-related mortality of patients. Therefore, more effective and safe therapeutic approaches for DHL are urgently needed.

Phosphatidylinositol-3-phosphate 5-kinase (PIKfyve), an endosomal lipid kinase, acts to control the cytoplasmic leaflet of endosomes via protein-lipid interactions between its FYVE domain and phosphatidylinositol-3-phosphate (PI3P), thus controlling the complex and distinct cellular functions [4]. It was also demonstrated that inhibition of PIKfyve by multiple approaches causes the endocytic uptake of fluid-filled vesicles. The multiple cytoplasmic vacuoles subsequently pinch off the plasma membrane in dividing cells, leading the cells burst as a result of copious “drinking” of extracellular fluid, which is termed as methuosis (a novel form of non-apoptotic cell death) [5, 6]. Recent studies indicated the therapeutic relevance of PIKfyve in multiple disease areas, including viral infections, neurodegenerative disorders, and cancers [7–10]. A highly selective and potent PIKfyve inhibitor, apilimod, successfully entered a clinical trial of patients with B-cell malignancies based on the superb activity in cancer cell lines and mouse models, as well as its well-tolerable property in the human body [11]. However, whether targeting PIKfyve would act as a potentially useful therapeutic approach for the tough DHL treatment still needs to be elucidated.

As the component of cytoskeletal fibers, microtubules, which are formed by two tubulin molecules (α-tubulin and β-tubulin), play crucial roles in lots of cellular functions, including cell division, cell mobility, and cell–cell interactions [12]. Therefore, interference with microtubule dynamics by tubulin inhibitors represented an attractive approach for cancer therapeutics. The most successful microtubule/tubulin inhibitor is vincristine [13], which has been the mainstay of lymphocytic leukemia treatment for many years. However, whether a tubulin inhibitor can be combined with a PIKfyve inhibitor for more aggressive lymphomas has not been explored.

In the pursuit of small molecules capable of triggering the novel type of cell death that may be useful for the development of therapeutics of current unmet clinic needs, we previously discovered the azaindole derivative, HZX-02-059, as a potent methuosis inducer, which showed both in vitro and in vivo anti-tumor activity against triple-negative breast cancer (TNBC) [14]. Then we further investigated the molecular target of the HZX-02-059 compound. In the present study, we reported that HZX-02-059 could act as a potent cytotoxic anti-tumor agent in DHL cells in vitro and in vivo as well as its molecular mechanisms of cell death induction. The beneficial dual-targeting effect rationalized the combination of PIKfyve and tubulin inhibitors for the treatment of DHL.

## Results

### HZX-02-059 is a PIKfyve and tubulin dual-target inhibitor

**Fig. 1:**
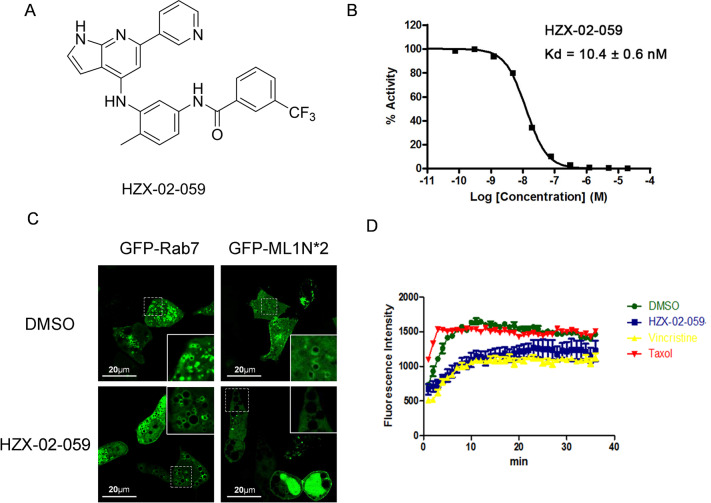
HZX-02-059 is a PIKfyve and tubulin dual-target inhibitor. **A** The structure of HZX-02-059. **B** HZX-02-059 has a high affinity for PIKfyve with *K*_d_ value of 10.4 ± 0.6 nM. Binding constant was detected by DiscoveRX Corporation, San Diego, CA. **C** HZX-02-059 reduced PI(3,5)P2 production and location on late endosome/lysosome membrane. 293T cells were transfected with GFP-Rab7 or GFP- ML1N*2 and then followed by 2 μM HZX-02-059 treatment for 8 h. DMSO was used as a control. **D** HZX-02-059 suppresses tubulin polymerization. Tubulin polymerization was performed in vitro using a tubulin polymerization assay kit (Cytoskeleton, cat# BK011P). 100 μM HZX-02-059 was used while Vincristine and Taxol were used as references with a concentration of 3 μM.

HZX-02-059 was identified as a methuosis inducer with strong vacuolation ability in our previous studies, but its target was unclear. During the further investigation of the molecule target of HZX-02-059, we profiled it against a broad panel of 468 kinases using KINOMEscan technology (DiscoverX, USA) [15]. The results indicated that the affinity against PIKfyve was very high, with a percent of control of 0.35% at a 1 µM (Supplemental Table S1). Other potential targets were also hit, such as BRAF(V600E), DDR1, DDR2, EPHA2, EPHA8, EPHB2, FRK, KIT, LCK, PDGFRB, and RAF1, if the cut-off was set at 5%. CSF1R, EPHA4, and TNNI3K were further found if the cut-off was set at 10%. The *K*_d_ value was further determined to be 10 nM (Fig. 1B), which suggests that PIKfyve is a potential target for HZX-02-059. We further used a genetically encoded fluorescent probe GFP-ML1N*2 to detect the direct lipid substrate phosphatidylinositol-3,5-bisphosphate (PI(3,5)P2) of PIKfvye [16]. As shown in Fig. 1C, Rab7 is a late endosome/lysosome marker. Upon treatment of HZX-02-059, Rab7 was accumulated on the membrane of enlarged vacuoles. GFP-ML1N*2 probe was also localized to the late endosome/lysosome membrane in the DMSO-treated cells. However, HZX-02-059 treatment dramatically reduced the membrane localization of the GFP-ML1N*2 probe, indicating the absence of PI(3,5)P_2_ resulting from the inhibition of PIKfyve. These data were consistent with another methuosis inducer MOMIPP, having a similar core chemical structure of indole [17]. Taken together, HZX-02-059 targets PIKfyve and blocks its kinase activity in vivo and in vitro. However, inhibition of PIKfyve alone cannot explain the methuosis-inducing capability of HZX-02-059. We, therefore, searched for other molecular targets and found it also inhibited tubulin (Fig. 1D). Taken together, we boldly hypothesized that HZX-02-059 was an effective dual-targeted inhibitor of PIKfyve and tubulin.

### HZX-02-059 exerts anti-proliferative action of DHL cells in a dose- and time-dependent manner

**Fig. 2:**
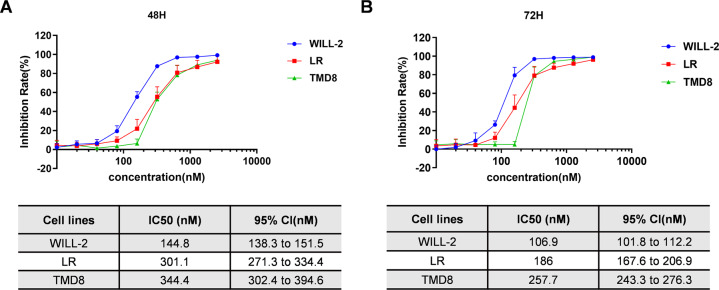
HZX-02-059 exerts anti-proliferative action of DHL cells in a dose- and time-dependent manner. Inhibition rate and IC_50_ values were shown after exposure to indicated concentration of HZX-02-059 for 48 h (**A**) and 72 h (**B**) in DHL cells by CCK-8 assay. Values indicate mean ± S.D. for at least three independent experiments performed in triplicate.

We then assessed the cytotoxic effect of HZX-02-059 against various human DHL cell lines, including Will-2, LR, and TMD8. Cells were treated with various concentrations of HZX-02-059 for 48 and 72 h, and cell viability was determined by CCK-8 assay. As shown in Fig. 2A, B, HZX-02-059 significantly inhibited the proliferation activity in all tested DHL cell lines in a dose- and time-dependent manner (all *P* < 0.05 for each dosage vs untreated control) while the sensitivity varied among these cell lines (Will-2 > LR > TMD8). Simultaneously, the IC_50_ values for each cell line at 48 and 72 h were also calculated and shown on the basis of cell viability.

### HZX-02-059 induces a caspase-independent cell death in DHL cells

**Fig. 3:**
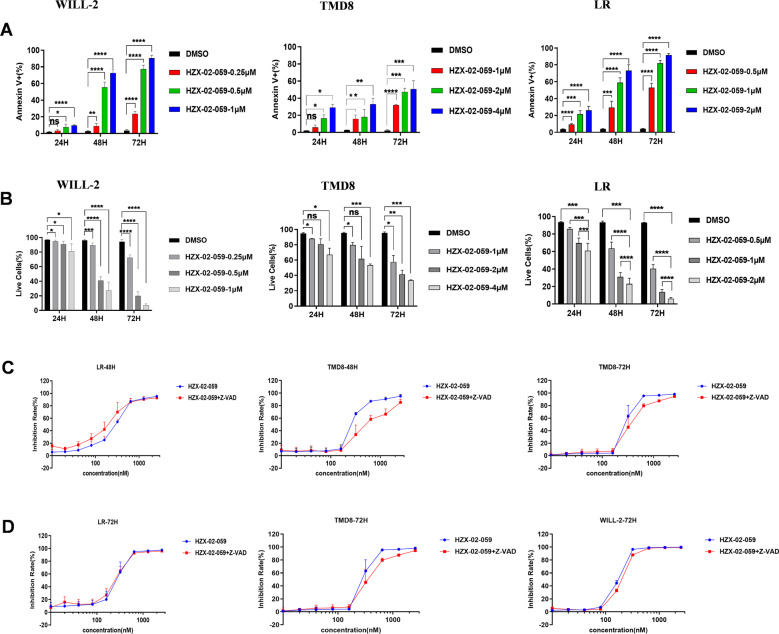
HZX-02-059 induces a caspase-independent cell death in DHL cells. **A**, **B** DHL cells were exposed to the indicated concentrations of HZX-02-059 for 24, 48, 72 h, after which percentages of apoptotic cells (**A**) and live cells (**B**) were determined by flow cytometry. **C**, **D** LR, TMD8, WILL-2 cells were pretreated with 20 μM Z-VAD-fmk for 2 h, followed by indicated concentrations of HZX-02-059 for an additional 48 h (**C**) and 72 h (**D**), and then the inhibition rates were assessed by CCK-8 assay. Values indicate mean ± S.D. for at least three independent experiments performed in triplicate. *P < 0.05, **P < 0.01, ***P < 0.001, ****P < 0.0001.

We further examined whether HZX-02-059 induces cell death in DHL cells. Diverse DHL cell lines were treated with indicated concentrations of HZX-02-059 for the indicated hours, and then subjected to flow cytometric analysis after Annexin V/PI staining. The percentage of dead cells (i.e., Annexin V^+^) and live cells (i.e., Annexin V^−^/PI^−^) were measured. As shown in Fig. 3A, B, administration with HZX-02-059 was able to induce a significant increase in the percentage of dead cells and a decrease in live cells in a dose-dependent manner in Will-2, LR and TMD8 cells (all *P* < 0.05 for each dosage vs untreated control in three cell lines at every time point). Besides, in addition to the marked increases in the percentage of cell death (Annexin V^+^), representative flow cytometric data (Supplemental Figs. 2–4) also showed that the HZX-02-059 treatment predominantly induced late apoptosis (Annexin V^+^/PI^+^, upper/right quadrant Q2), rather than early apoptosis (Annexin V^+^/PI^−^, lower/right quadrant Q3) nor necrosis (Annexin V^−^/PI^+^, upper/left quadrant Q1).

Given the important effect of caspases in the execution program of apoptosis, we wondered whether HZX-02-059-induced cell death still depended on the activation of caspases. To this end, Z-VAD-fmk, a broad-spectrum caspase inhibitor, was employed in this study. As shown in Fig. 3C, D, co-culture with Z-VAD-fmk was unable to salvage the HZX-02-059-induced cell death (Red lines, all *P* > 0.05 for each dosage vs DMSO control), suggesting that HZX-02-059 induces a caspase-independent cell death.

### HZX-02-059 triggers non-apoptotic cell death of methuosis as a result of PIKfyve/ TFEB inhibition

**Fig. 4:**
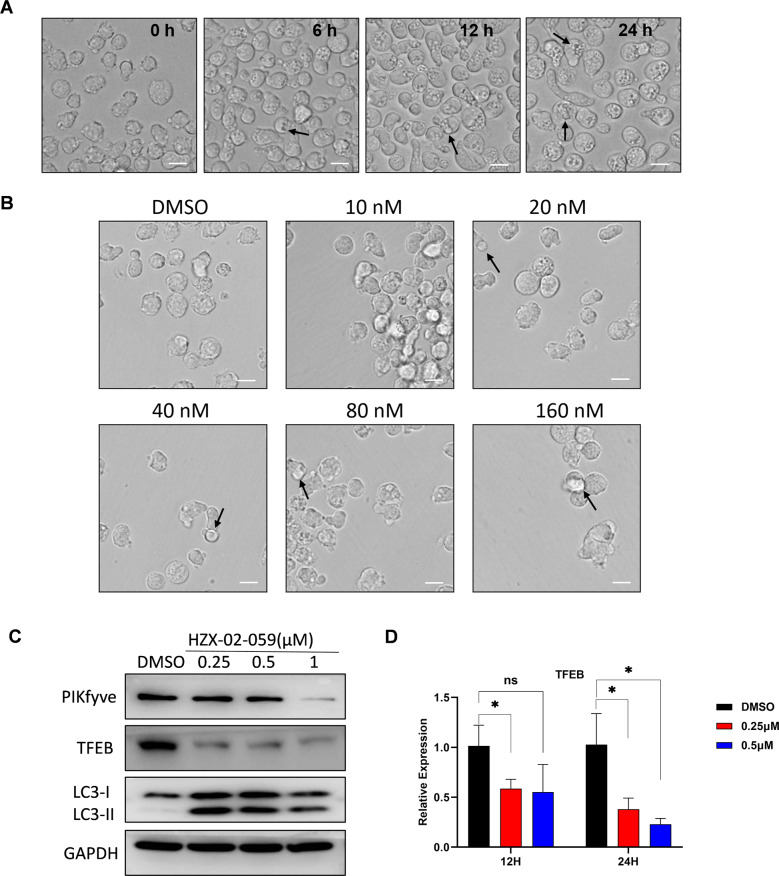
HZX-02-059 triggers non-apoptotic cell death of methuosis as a result of PIKfyve/TFEB inhibition. **A** Morphological changes in cytoplasm and irregular variations of the cell membrane after 6, 12, 24 h treatment with 2 μM HZX-02-059 using an optical microscope. Scale bars = 20 μm. **B** Intracellular vesicles were not degraded until cell death even though exposed to nanomole concentration of HZX-02-059 for consecutive 5 days. **C** Protein levels of LC3, PIKfyve, and TFEB were evaluated by western blotting after the drug’s administration. GAPDH was served as a loading control. **D** Real-time PCR analysis was performed to validate the expression of TFEB in Will-2 cells treated with HZX-02-059 for 12 h and 24 h. The reaction was carried out in triplicate and relative expression levels were calculated as 2^−^^△△CT^ after normalization to β-actin. Values indicate mean ± S.D. for at least three independent experiments performed in triplicate (*P < 0.05).

Further evaluation of the HZX-02-059-treated DHL cells under microscopy was also conducted. As shown in Fig. 4A, administration with 2 μM HZX-02-059 for 6, 12, 24 h in Will-2 cells revealed more, bounded, large and empty vacuoles compared to the DMSO control, which in accord with the characteristics of methuosis [6, 18, 19]. Moreover, intracytoplasmic vesicles can still be observed and were not degraded until cell death even though exposed to a low concentration of HZX-02-059 (e.g., nanomolar level) for a long time (Fig. 4B).

Western blots for PIKfyve expression were also carried out. As shown in Fig. 4C, HZX-02-059 treatment lowered the expression of PIKfyve to the same extent. Recently, PIKfyve/transcription factor EB (TFEB) signaling axis was identified as a determinants pathway in NHLs to affect the acidification of the lysosomal compartment and endolysosomal membrane trafficking, thus disrupting autophagy activity [20]. It is widely known that TFEB acts as a master regulator of the function of lysosomes and autophagy [21, 22]. Hence, we also assessed the expression of TFEB and autophagy-relevant LC3 I/II upon the HZX-02-059 treatment. A significant decline in the level of TFEB and significant conversation of LC3 I/II was obviously observed (Fig. 4C), supporting the disorderliness of lysosomes and autophagy. In addition, we also found that the reduction of TFEB undergoing HZX-02-059 exposure occurred as early as 12 h as shown in Fig. 4D.

### HZX-02-059-induced cell cycle arrest is mechanistically associated with the inhibition of tubulin and mTOR/Myc axis

**Fig. 5:**
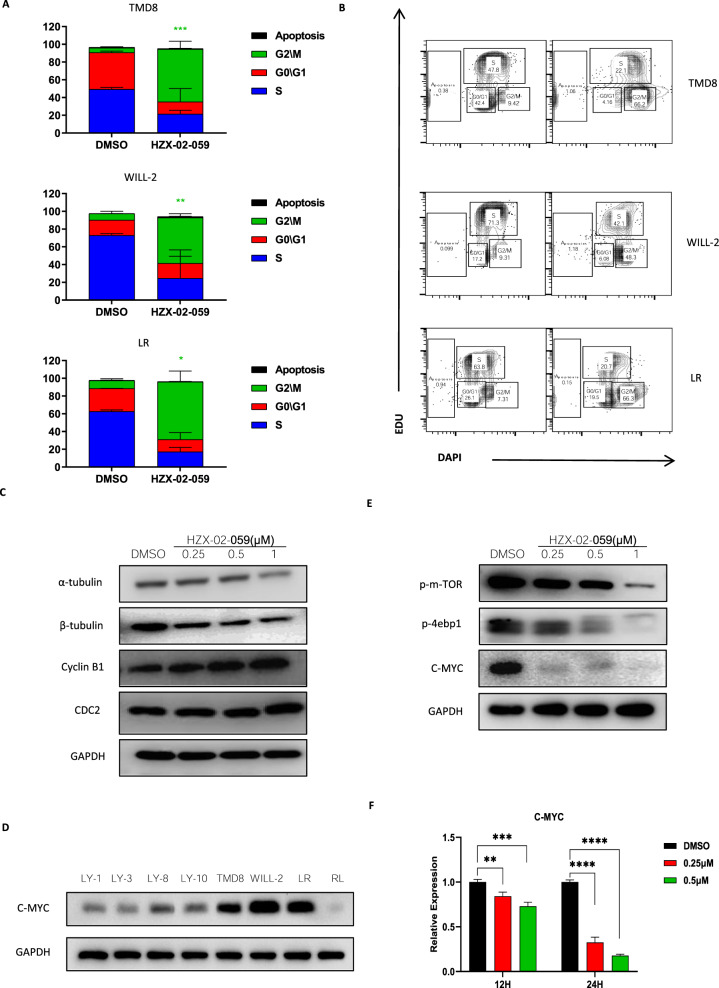
HZX-02-059-induced cell death is also mechanistically associated with mTOR–Myc axis. **A**, **B** DHL cell lines were exposed to 0.5 μM HZX-02-059 for 24 h, after which cell cycle status was determined by FACS. Representative data (**A**) for flow cytometric analysis and histogram (**B**) were shown. **C** Cell cycle-related proteins, including Cyclin B1, Tubulin, and CDC2, were assessed by western blot analysis after drug exposure. **D** Expression level of MYC between DCLBC and DHL cell lines. **E** Protein levels in the mTOR/MYC signaling axis were also evaluated. GAPDH was served as a loading control. **F** Transcriptional level of C-MYC after HZX-02-059 treatment. Values indicate mean ± S.D. for at least three independent experiments performed in triplicate *P < 0.05, **P < 0.01, ***P < 0.001.

It is well established that tubulin-destabilizing agents induced the cell cycle arrest at the G2/M phase, which attributed to the microtubule depolymerization and cytoskeleton disruption [23]. This prompted us to evaluate the effect of HZX-02-059 on cell cycle distribution. As shown in Fig. 5A, the cell cycle arrest was obviously observed. Treatment with 0.5 μM HZX-02-059 for 24 h in DHL cells resulted in a significant decrease of the percentage of cells in the S phase and G0/G1 phase, and a remarkable increase in G2/M phase in all tested cell lines. Representative data for flow cytometric analysis of cell cycle was shown in Fig. 5B. The effect of HZX-02-059 on tubulin and cell cycle regulatory proteins was also determined to further identify the effect of cell cycle arrest. As shown in Fig. 5C, HZX-02-059 treatment in WILL-2 cell line effectively inhibited the expression of α-tubulin and β-tubulin, as well as the Cyclin B1 and CDC2, which were associated with the cell cycle arrest.

Earlier studies reported that double-hit lymphoma harbored MYC translocations or rearrangement, which led to high expression of MYC. MYC acts as a protooncogene and plays an important role in cancer cell growth, division and metabolism. As shown in Fig. 5D, the expression of MYC was higher in double-hit lymphoma than in the other non-DHL lymphoma cell lines. Then, the transcription and translation of MYC were assessed after HZX-02-059 treatment in WILL-2 cell line. The results showed the c-Myc level was tremendously abrogated after administration with HZX-02-059 (Fig. 5E, F). The mammalian target of rapamycin (mTOR) has emerged as a critical effector in cell signaling pathways commonly dysregulated in human cancers, and is well-known involved in the regulation of cell cycle [24, 25]. Therefore, we also investigated the role of HZX-02-059 on the mTOR signaling pathway, which is one of the upstream pathways of c-MYC. As shown in Fig. 5D, exposure to HZX-02-059 resulted in a robust decrease in the phosphorylated level of mTOR and its downstream 4EBP1. Taken together, these results demonstrated that the treatment of HZX-02-059 inhibited mTOR signaling and its downstream targets c-MYC, thereby preventing the growth of malignant DHL cells.

### HZX-02-059 suppresses growth of xenograft derived from DHL cells with a low/non-systemic toxicity profile

**Fig. 6:**
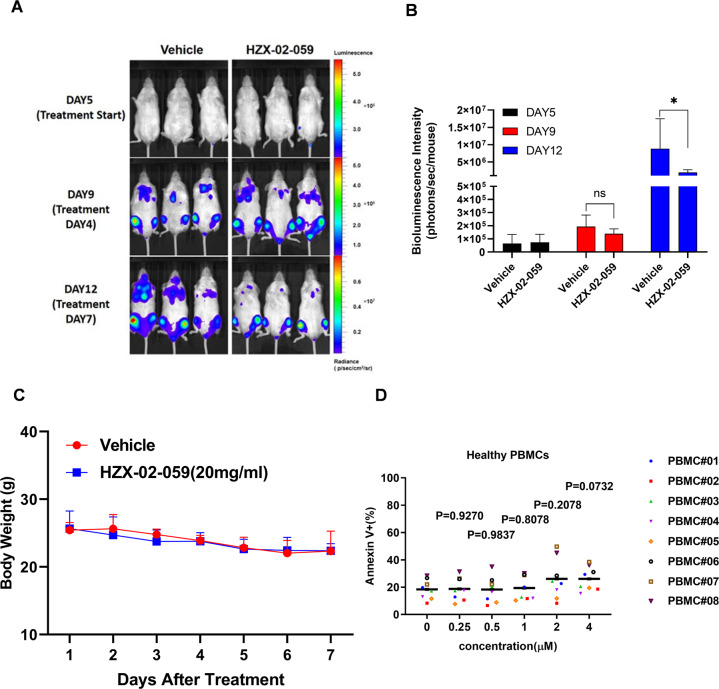
HZX-02-059 suppresses growth of xenograft derived from DHL cells with a low systemic toxicity profile. **A** Serial bioluminescence images of mice-bearing luciferase-labeled LR tumors were shown after treated with vehicle or HZX-02-059 (20 mg/kg) by intraperitoneal injection. **B** Quantification of bioluminescence emitted from the whole body of each mouse in Fig. 5A. **C** Body weight changes between vehicle and HZX-02-059 treatment. **D** HZX-02-059 treatment spared normal hematopoietic cells derived from peripheral blood of 9 healthy donors.

Next, to evaluated the in vivo anti-tumor efficacy of HZX-02-059, an established xenograft mouse model was used. The xenograft model was generated by injection of luciferase-labeled LR cells into NOD-Prkdc^−/−^IL2rg^−/−^ (NPI) mice followed by 1 Gy irradiation as described in detail in Methods. After confirmation of engraftment by bioluminescence imaging (BLI) at day 5, mice were randomly assigned to two groups and received vehicle or 20 mg/kg HZX-02-059 treatment by intraperitoneal injection for consecutive 7 days. BLI was used to monitor the tumor burden at multiple time points. As shown in Fig. 6A, B, mice who received HZX-02-059 treatment displayed a significant inhibition of tumor growth after 7 days’ treatment (*P* < 0.05 at Day 12 vs vehicle group). However, there was no statistically significant difference in the average body mass of mice between these two groups (Fig. 6C), revealing the high efficacy but low toxicity anti-tumor action of HZX-02-059 in vivo. We also examined the activity of HZX-02-059 in normal primary samples (*n* = 9) from peripheral blood mononuclear cells (PBMCs). As shown in Fig. 6D, treatment with various concentrations of HZX-02-059 for 72 h did not induce apoptosis in healthy donor PBMCs (all *P* > 0.05 for each dosage vs DMSO control). These findings argue that HZX-02-059 might act as an effective anti-tumor agent with only minor/non-toxicity in the treatment of DHL.

### PIKfyve inhibitor (apilimod) combined with tubulin inhibitor (vincristine) exerts drastic synergistic cytotoxicity toward DHL cells

**Fig. 7:**
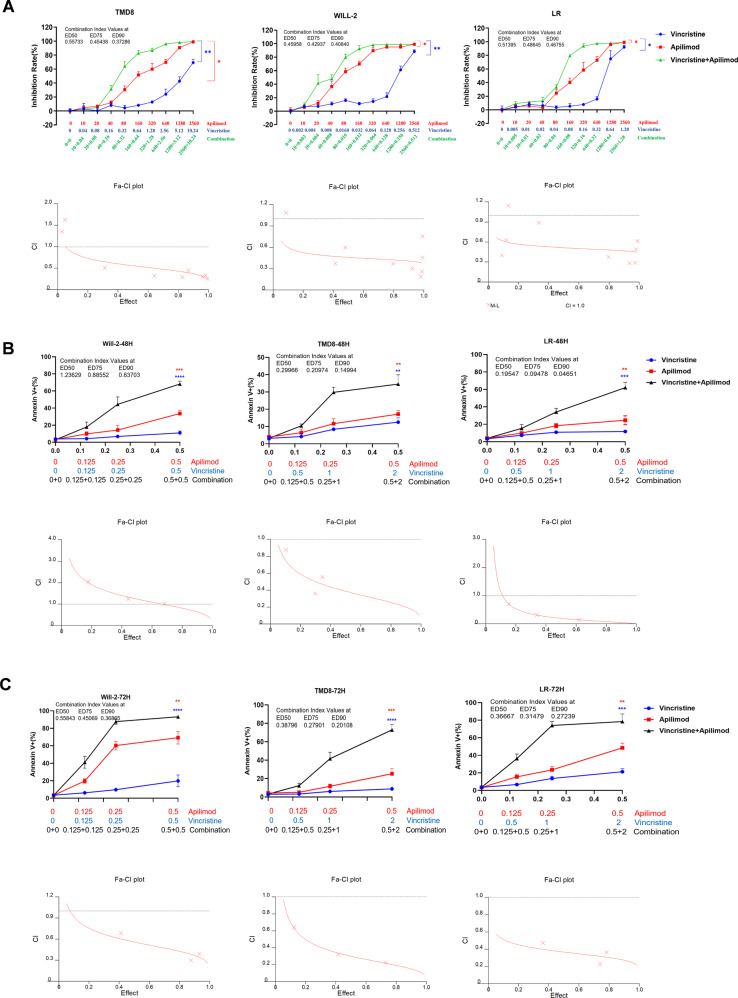
PIKfyve-specific inhibitor (apilimod) combined with tubulin-specific inhibitor (vincristine) exerts drastic synergistic cytotoxicity toward DHL cells. Anti-proliferative effect (**A**) and apoptotic cells (**B**, **C**) were determined after LR, WILL-2, and TMD8 cells treated with indicated concentrations of PIKfyve-specific inhibitor apilimod and tubulin-specific inhibitor vincristine for 72 h or 48 h. Combination index plot were also shown based on the inhibition rates or Annexin V-positive cells. CI < 1 was defined as synergism. Values indicate mean ± S.D. for at least three independent experiments performed in triplicate. *P < 0.05, **P < 0.01, ***P < 0.001, ****P < 0.0001.

Last, in order to better illustrate the anti-tumor activity of PIKfyve and tubulin dual-target inhibitor HZX-02-059, PIKfyve-specific inhibitor apilimod and tubulin-specific inhibitor vincristine were employed in this study to examine whether the combination would have a synergistic effect on DHL cells. As shown in Fig. 7A, treatment with apilimod and vincristine alone significantly inhibited the cell growth of Will-2 in a dose-dependent manner, while the combination led to markedly enhanced growth inhibition. Combination index (CI), the method used for the synergy quantification of drug-drug interaction, was also calculated. Strong synergism effects were observed as indicated by CI values at ED_50_ /ED_75_ /ED_90_ < 1.

The synergistic effect of the combination was further confirmed by apoptosis assay. As shown in Fig. 7B, C, treatment with apilimod and vincristine alone only induced slight cell death, while the percentage of dead cells was dramatically increased after co-exposure to the combined treatment. Furthermore, a strong synergism effect was also observed, evidenced by all CI values < 1. Notably, representative flow cytometric data (Supplemental Figs. S5–7) indicated that the combined treatment still predominantly induced late apoptosis (Annexin V^+^/PI^+^, upper/right quadrant Q2), which is in line with the effect of HZX-02-059 (Supplemental Figs. S2–4).

## Discussion

The most common lymphoma type of DHL is diffuse large B-cell lymphoma (DLBCL) with t(14;18) and t(8;14) or t(8;22) translocation, leading to the overexpression of BCL-2 and MYC, respectively. The overexpression of MYC generates the uncontrolled proliferation ability, while BCL-2 renders the cells lose the brake for apoptotic responses, which both contribute to a perfect storm for carcinogenesis [26, 27]. Increasing evidences suggest that patients with DHL are more likely to experience poor survival under the standard treatment of R-CHOP [28]. Therefore, optimizing alternative therapeutic strategies for patients with DHL, especially those with more lactate dehydrogenase (LDH), higher international prognostic index (IPI) scores, and additional infiltration, are urgently needed. In this pre-clinical study, we firstly confirm the cytotoxicity activity of HZX-02-059, a novel PIKfyve and tubulin dual-target inhibitor, against DHL cell lines in vitro and in vivo, which might further provide evidence that HZX-02-059 might act as a potential candidate agent for the treatment of DHL in the near future.

In the present study, in order to identify HZX-02-059-induced cytotoxicity in DHL cells, we conducted the cell death analysis by co-staining Annexin V (a marker for early apoptosis) and 7-AAD (a membrane-impermeable marker of nonviable cells) after drug exposure. We observed a significant increase in the percentage of cells with Annexin V^+^/PI^+^ which could be indicative of late apoptosis (Supplemental Figs. S2–4). However, we also found the precise mechanism of HZX-02-059-induced cell death might not be the canonical caspase-dependent apoptosis as the cell death caused by HZX-02-059 cannot be prevented by pre-treatment with pan-caspase inhibitor (Fig. 3C, D). Moreover, we observed a marked conversion of LC3I/II, suggesting that HZX-02-059 appeared to disrupt the completion of autophagy. Nevertheless, these primitive findings remain to be confirmed in our success investigations.

The conception of programmed cell death has been addressed for almost 50 years. One of the most widely accepted and classic way of cell death is intrinsic and/or extrinsic apoptosis, which is characterized by cell shrinkage, nuclear chromatin condensation, nuclear chromosome fragmentation, and caspase activation [29, 30]. Thus, apoptosis is often regarded as caspase-dependent cell death. Distinct from apoptosis, HZX-02-059-induced cell death ensues as the vacuoles expand to fill most of the cytoplasmic space and cell membrane integrity is disrupted (Figs. 4A, B). And it is caspase-independent, evidenced by Fig. 3. Besides, our study demonstrated that the HZX-02-059-induced cell death of methuosis was a result of PIKfyve/TFEB inhibition.

Matthew et.al. reported the first phase I clinical trial of venetoclax, a novel Bcl-2 selective inhibitor, against patients with relapsed or refractory Non-Hodgkin Lymphoma (R/R NHL) [31]. In their work, the efficacy of venetoclax monotherapy varied among NHL subtypes. Of note, the overall response rate of DLBCL was 18%, while the estimated median progression-free survival of DLBCL was only 1 month. Therefore, targeting Bcl-2 molecular does not seem to obtain a satisfactory therapeutic effect toward relapsed or refractory DLBCL. Considering that the Bcl-2-specific inhibitor venetoclax has no significant clinical efficacy, it is suggested that MYC might play a major driving role in the mechanism of the dismal outcome of DHL. However, a large number of pharmacological studies directly targeting MYC genes have no substantial progress, frontier research mainly focused on the key molecules and signaling that regulate *Myc* gene. In this study, the expression of Myc was significantly blocked by HZX-02-059-induced mTOR signaling inhibition, suggesting that HZX-02-059 might serve as a potential agent for the treatment of DHL with Myc rearrangement.

Last, the PIKfyve-specific inhibitor apilimod and tubulin-specific inhibitor vincristine were employed to mimic the dual-target inhibitor HZX-02-059. The combination of apilimod with vincristine acted with a strong synergistic effect on killing DHL cells, which also indirectly illustrated the reason for the significant anti-tumor effect of HZX-02-059 with dual targets. These data highlight the great potential for developing dual inhibitor against both PIKfyve and tubulin.

The limitation of this study only includes the cell line xenograft model which lacks predictive value with regard to clinical outcome in specific cancer types. For that reason, our subsequent studies will focus on the in vivo efficacy of HZX-02-059 against diverse patient-derived models, which has been proven to be the most sensitive predictors of clinical responses in patients and offers the potential for personalizing patient cancer treatment.

## Conclusions

In summary, our study demonstrated that HZX-02-059, a novel PIKfyve and tubulin dual-target inhibitor, exerts significant anti-tumor actions in diverse DHL cell lines in vitro and in vivo without apparent side effects. Our findings proved initial evidence for further evaluation of HZX-02-059 as an effective therapeutic approach in the treatment of DHL.

## Materials and methods

### Synthesis of HZX-02-059

The synthesis of HZX-02-059 was depicted in our previous work. ^1^H NMR (600 MHz, DMSO-*d*_6_) δ 11.48 (s, 1H), 10.46 (s, 1H), 9.09 (d, *J* = 2.2 Hz, 1H), 8.52 (d, *J* = 4.8 Hz, 1H), 8.34 (s, 1H), 8.27 (s, 1H), 8.24 (dd, *J* = 12.9, 8.0 Hz, 2H), 7.95 (d, *J* = 7.8 Hz, 1H), 7.87 (d, *J* = 2.2 Hz, 1H), 7.77 (t, *J* = 7.8 Hz, 1H), 7.57 (d, *J* = 8.0 Hz, 1H), 7.43 (dd, *J* = 7.9, 4.7 Hz, 1H), 7.34 (d, *J* = 8.3 Hz, 1H), 7.22 (t, *J* = 2.9 Hz, 1H), 6.77 (s, 1H), 6.53 (t, *J* = 2.7 Hz, 1H), 2.24 (s, 3H), MS (ESI) *m*/*z* [C_27_H_20_F_3_N_5_O + H]^+^. See Supplemental Fig. 1 for the HPLC spectrum.

### Cell lines and cell culture

DHL cell lines WILL-2, LR, TMD8 were kindly provided by Prof.KH Young (Duke University, USA). Cells were cultured in RPMI-1640 (HyClone, Thermo Scientific, Waltham, MA, USA) supplemented with 10% FBS (FBS, PAN Seratech, Aidan Bach, Germany) and 1×P/S (HyClone) in a 37 °C incubator with 5% CO_2_. All cells were commercially authenticated by short tandem repeats genotyping (Shanghai Tissuebank Diagnostics Co., LTD, China) in July 2020 and tested for the presence of mycoplasma using the PCR method every other month.

### Primary samples

Normal peripheral blood cells were obtained from healthy donors for hematopoietic stem cell transplantation (*n* = 9) with informed consent. This study is carried out in accordance with the Declaration of Helsinki, and approved by the Ethics Committee Board of the First Affiliated Hospital of Xiamen University.

### Cell viability assay

In brief, cells were plated into 96-well plates with 1 × 10^5^/well containing 100 μl of growth medium and then treated with designated drugs for an appropriate time. The absorbance at 450 nm was read by a VERSA max microplate reader (Molecular Devices, Sunnyvale, CA, USA) after CCK-8 agents (MedChemExpress, New Jersey, USA) were added and further cultured for an additional 2 h. All experiments were repeated three times and performed in triplicate in each experiment. The IC_50_ values were calculated using Calcusyn software [32].

### Apoptosis assay

DHL cells were treated with designated drugs for indicated times and then harvested and subjected to flow cytometric analysis using Novocyte (ACEA Bioscience, San Diego, CA, USA) after Annexin V-FITC/PI (eBioscience, San Diego, California, USA) staining following the manufacturer’s instruction. All experiments were performed in triplicate, and the data were indicated as mean ± SD.

### Cell cycle analysis

In brief, drug-treated DHL cells were incubated with 10 µM 5-ethynyl-2′-deoxyuridine (EdU) for 2 h prior to fixation and permeabilization and then subjected to flow cytometric analysis using Click-iT EdU Alexa Fluor 647 Flow Cytometry Assay Kit (Thermo Fisher Scientific, USA) according to the manufacturer’s instructions. DNA content was staining with DAPI. All experiments were performed in triplicate, and the data were indicated as mean ± SD.

### Western blot analysis

Cell lysates (30 μg protein/lane) were electrophoresed in 10% SDS-PAGE and transferred to a PVDF membrane (Millipore, Billerica, MA, USA). The transblotted membranes were blocked for 1 h with 5% non-fat milk in TBS-T and then probed with primary antibodies overnight at 4 °C, followed by appropriate secondary HRP-conjugated antibody (Merck Millipore, USA). The immunobands were visualized using the Amersham Imager 600 (AI600, GE Healthcare, Chicago, USA) followed by an enhanced ECL substrate kit (GE Healthcare, Chicago, USA).

### In vivo tumor model and therapeutic study

Six-week-old female NOD-Prkdc^−/−^IL2rg^−/−^ mice (NPI, IDMO ltd., Beijing, China) were housed under pathogen-free conditions according to the animal care guidelines. The protocols for the animal studies were approved by Xiamen University Animal Care and Use Committees. In brief, six mice were injected with luciferase-labeled LR cells via a tail vain injection after receiving 1 Gy irradiation. After confirmation of engraftment by bioluminescence imaging (5 days after injection), mice were randomly assigned to two groups and treated with vehicle or HZX-02-059 (20 mg/mL) for consecutive seven days. The tumor burden of mice was monitored by a bioluminescence imaging system at multiple time points after intraperitoneal injection with luciferase substrate D-luciferin (Gold Biotechnology, St. Louis, MO, USA).

### Statistical analysis

Data were represented as mean ± S.D. of at least three independent experiments. Comparisons between two groups were analyzed using the 2-tailed Student’s *t*-test by GraphPad Prism software. *P* values < 0.05 were considered statistically significant.

## Supplementary information

Supplement figure1-7

Supplemental Table S1

## Data Availability

The data generated and/or analyzed during the current study are available from the corresponding author upon reasonable request.
