# Supplementary material for: Pharmacological targeting PIKfyve and tubulin as an effective treatment strategy for double-hit lymphoma
Source: Cell Death Discov. 2022 Jan 28;8:39. doi: 10.1038/s41420-022-00833-9 (PMC8799717; doi:10.1038/s41420-022-00833-9)

# HPLC spectrum of HZX-02-059

**Instruments**: Waters 2767 sample manager, Waters 2545 binary gradient module, Waters 2489 UV/Visible detector.
**Column**: SunFire$^{TM}$ C18 column, 5 µM, 4.6 x 50 mm.
**Solvent A**: methanol with 0.035% trifluoroacetic acid.
**Solvent B**: water with 0.035% trifluoroacetic acid.
**Wavelength**: 254 nm.

| Time (min) | Flow (mL/min) | A% | B% |
|---|---|---|---|
| 0 | 1 | 5 | 95 |
| 0.5 | 1 | 5 | 95 |
| 4.5 | 1 | 95 | 5 |
| 5.5 | 1 | 100 | 0 |
| 9 | 1 | 100 | 0 |
| 9.5 | 1 | 5 | 95 |
| 10 | 1 | 5 | 95 |

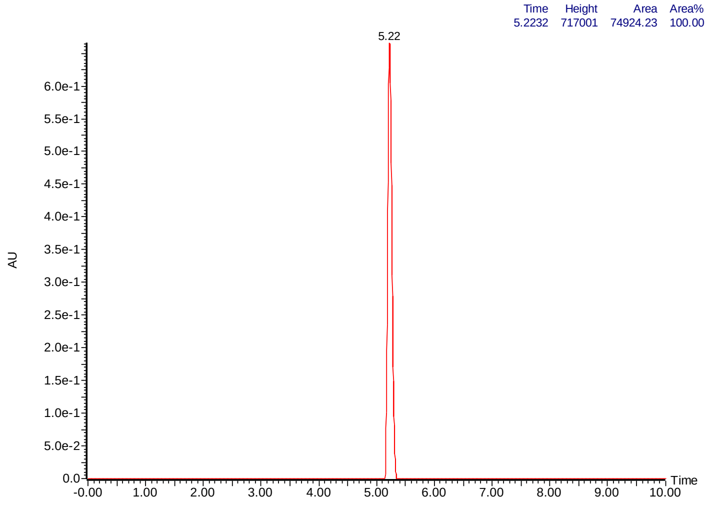


**Supplemental Figure S1**. HPLC spectrum of HZX-02-059.

# Supplemental Figure S2

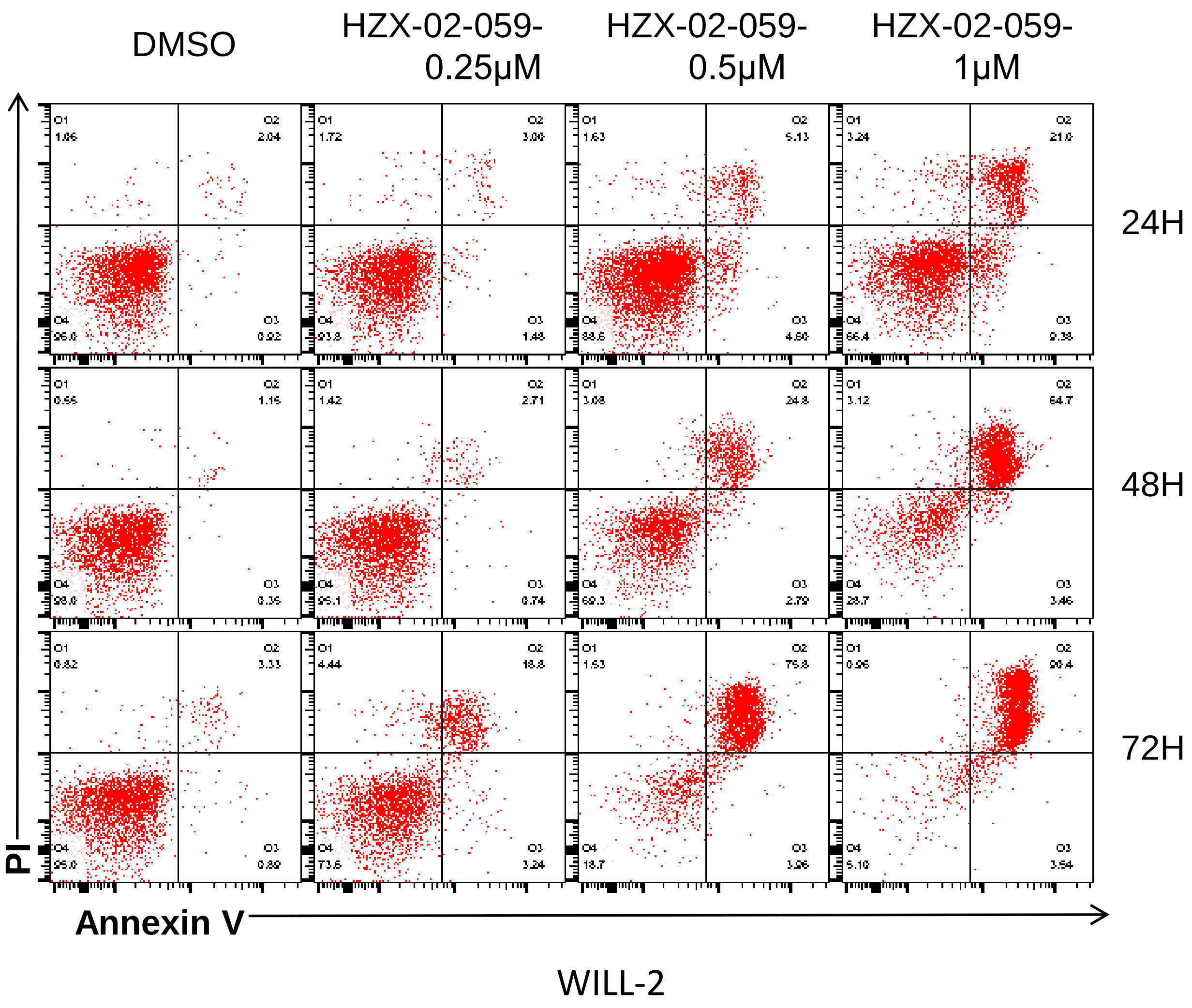


**Supplemental Figure S2-4.** Representative flow cytometric data for Annexin V/PI staining in LR, TMD8, WILL-2 cells after HZX-02-059 treatment for 24, 48, 72 hours.

# Supplemental Figure S3

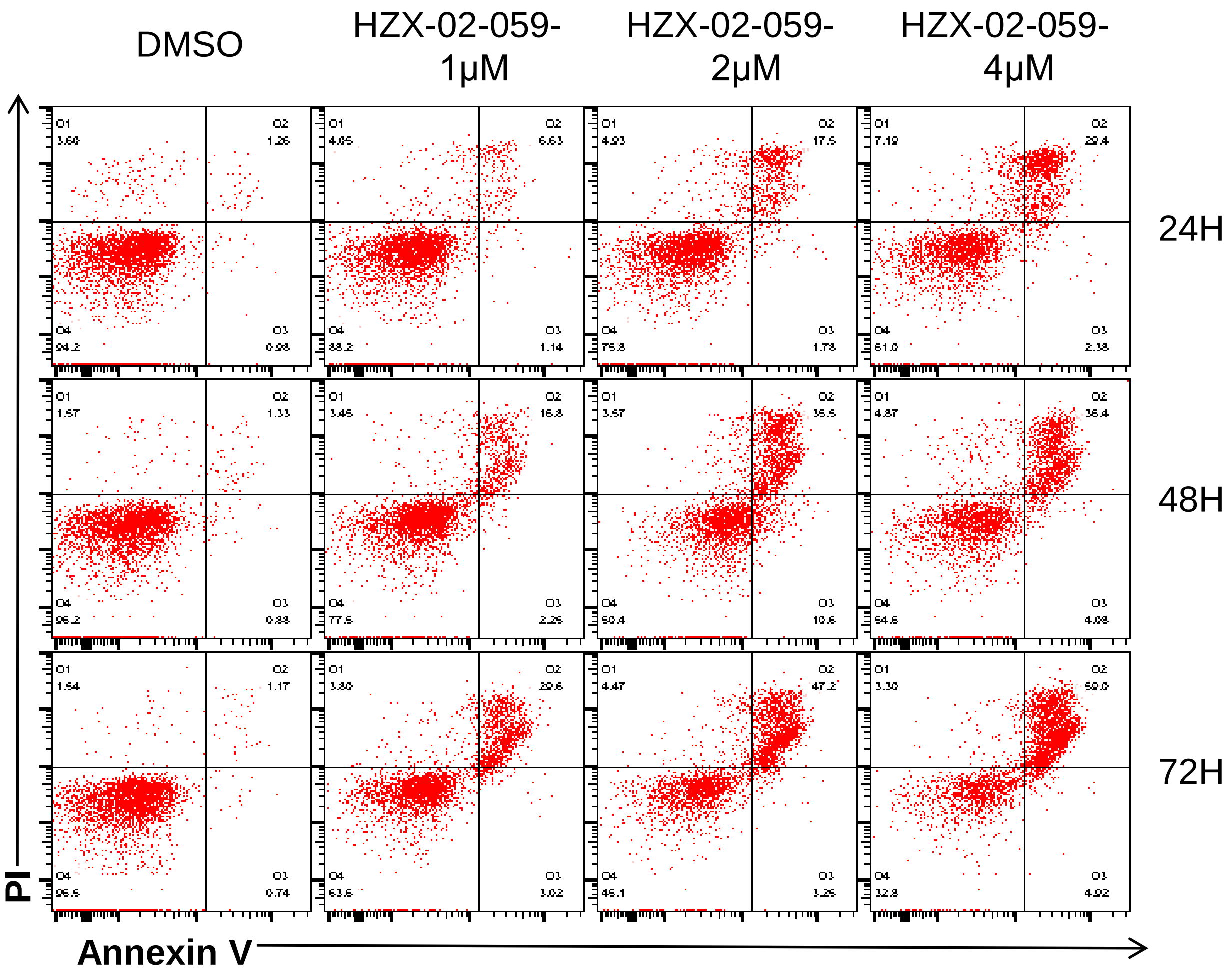


LR

# Supplemental Figure S4

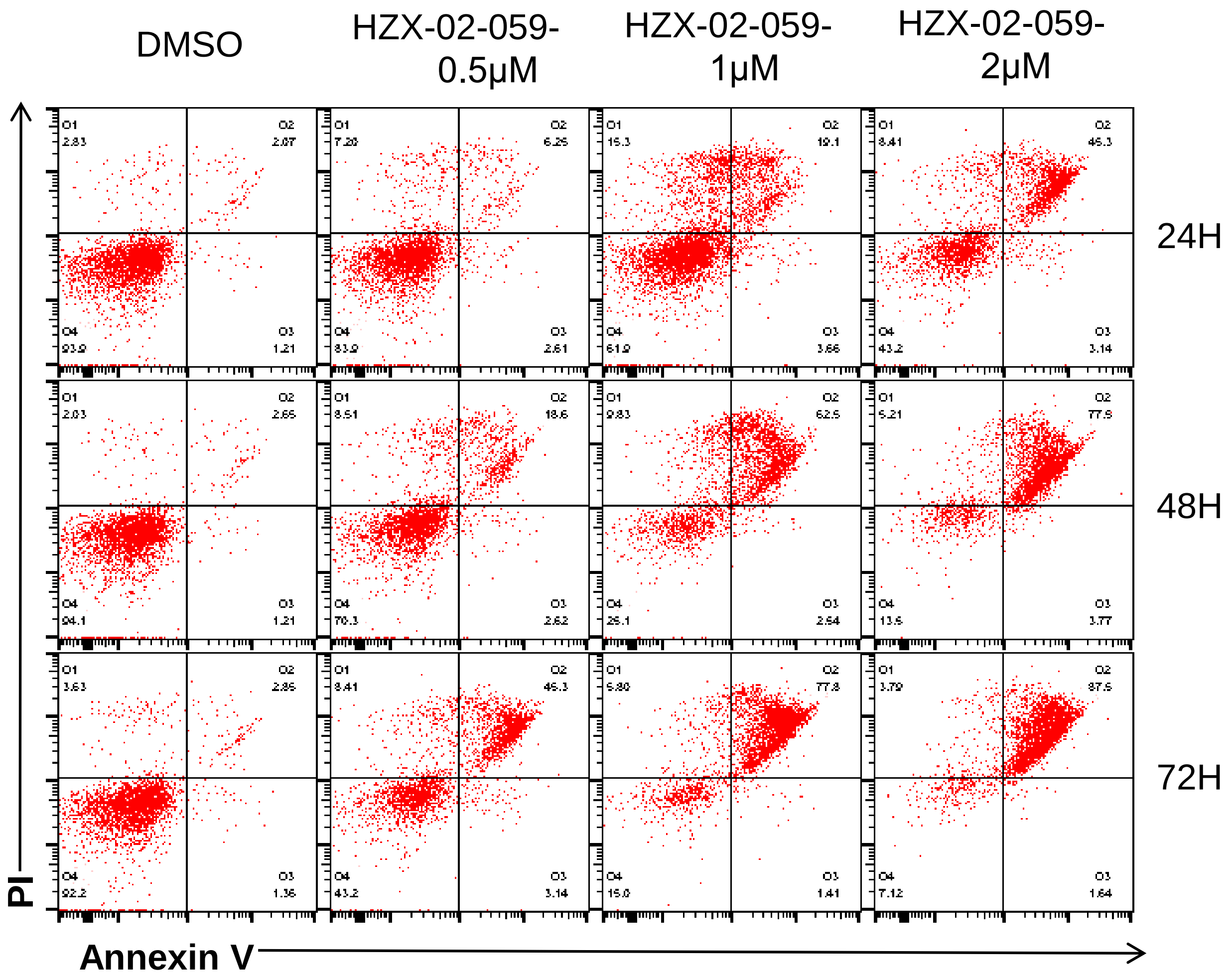


TMD8

# Supplemental Figure S5

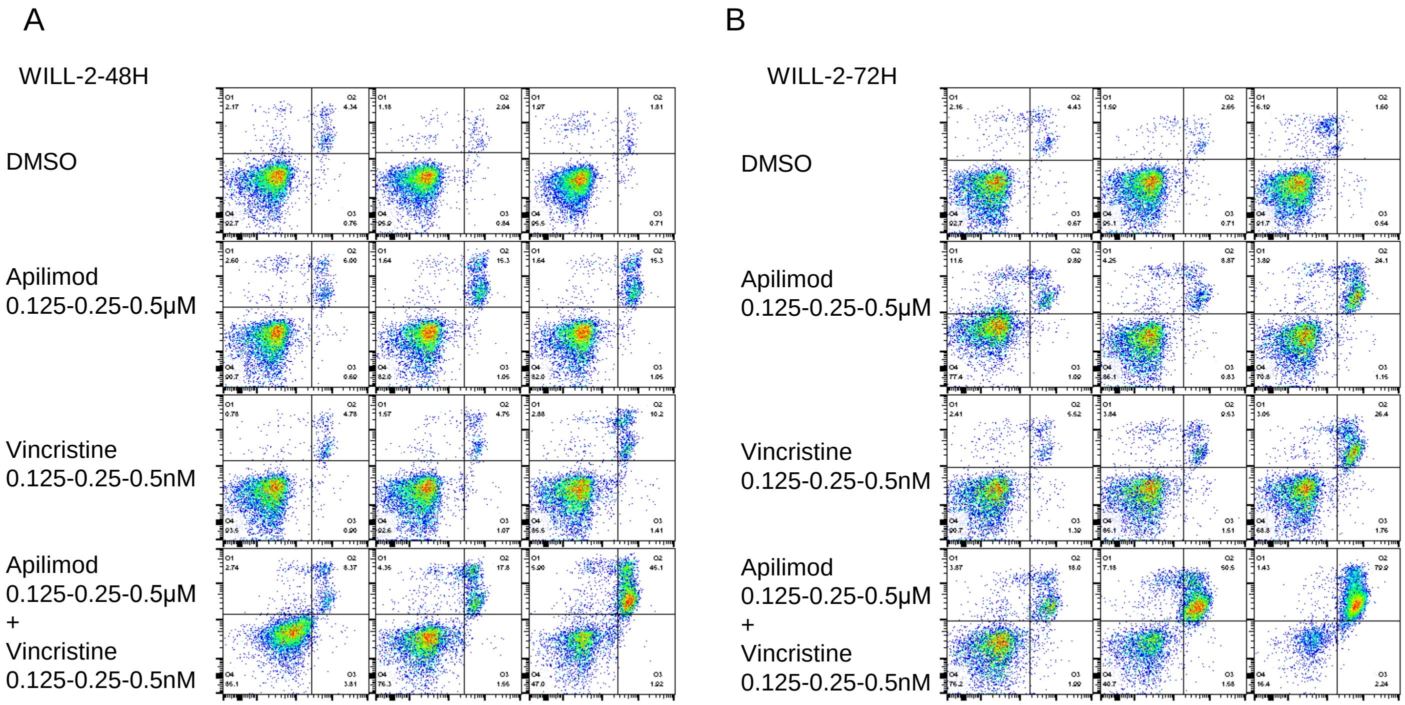


**Supplemental Figure S5-7.** Representative flow cytometric data for Annexin V/PI staining in LR, TMD8, WILL-2 cells after apilimod, vincristine or the combination treatment for 48, 72 hours.

# Supplemental Figure S6

A

TMD-8-48H

DMSO

Apilimod
0.125-0.25-0.5μM

Vincristine
0.5-1-2nM

Apilimod
0.125-0.25-0.5μM
+
Vincristine
0.5-1-2nM

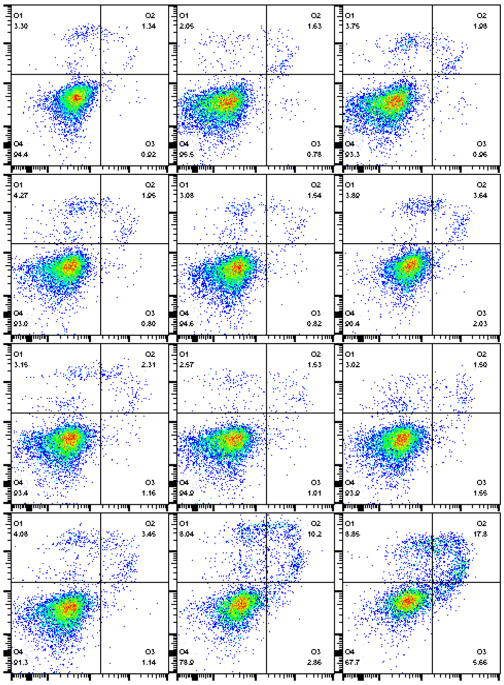

B

TMD-8-72H

DMSO

Apilimod
0.125-0.25-0.5μM

Vincristine
0.5-1-2nM

Apilimod
0.125-0.25-0.5μM
+
Vincristine
0.5-1-2nM

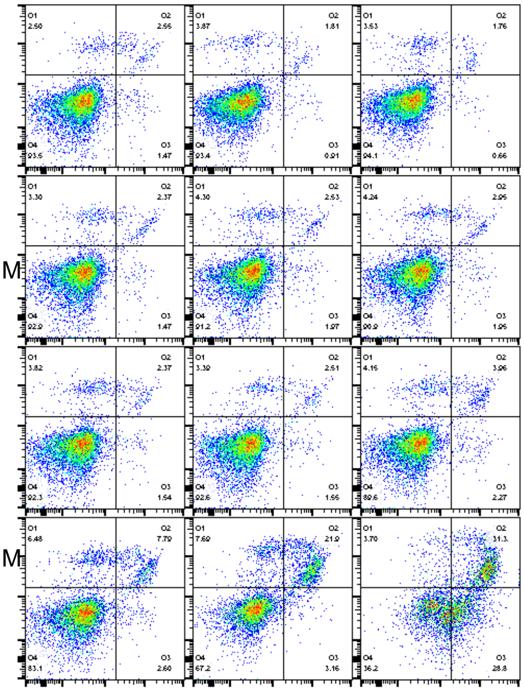

# Supplemental Figure S7

A

LR-48H

DMSO

Apilimod
0.125-0.25-0.5µM

Vincristine
0.5-1-2nM

Apilimod
0.125-0.25-0.5µM
+
Vincristine
0.5-1-2nM

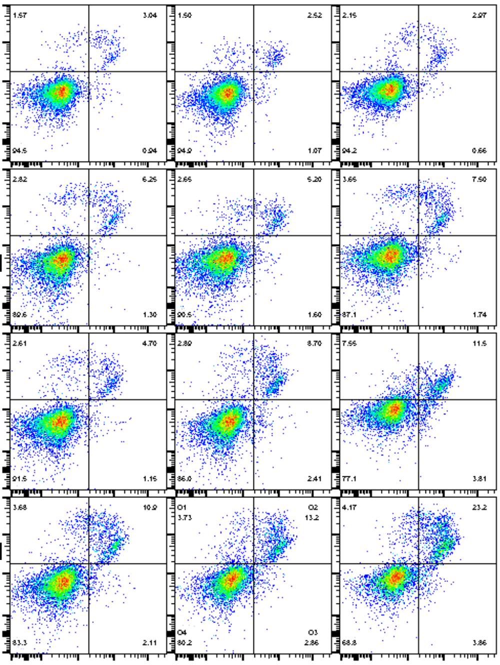

B

LR-72H

DMSO

Apilimod
0.125-0.25-0.5µM

Vincristine
0.5-1-2nM

Apilimod
0.125-0.25-0.5µM
+
Vincristine
0.5-1-2nM

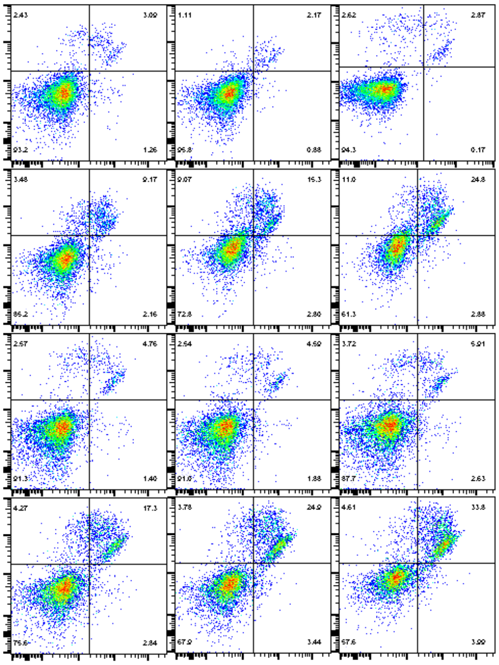

Supplement: Supplementary file 1 — Supplement figure1-7 [file 41420_2022_833_MOESM1_ESM.pdf]
